# The pathogenic E139D mutation stabilizes a non-canonical active state of the multi-domain phosphatase SHP2

**DOI:** 10.1101/2025.07.02.662799

**Published:** 2025-07-04

**Authors:** Anne E. van Vlimmeren, Ziyuan Jiang, Deepti Karandur, Anya T. Applebaum Licht, Neel H. Shah

**Affiliations:** 1 Department of Chemistry, Columbia University, New York, NY 10027; 2 Department of Biological Sciences, Columbia University, New York, NY 10027; 3 Independent researcher; 4 Current affiliation: Monarch Crops, Oakland, California, 94601; 5 Herbert Irving Comprehensive Cancer Center, Columbia University, New York, NY 10032

**Keywords:** *PTPN11*, tyrosine phosphatase, conformational ensemble, AlphaFold2, molecular dynamics

## Abstract

Dysregulation of the phosphatase SHP2 is implicated in various diseases, including congenital disorders and cancer. SHP2 contains two phosphotyrosine-recognition domains (N-SH2 and C-SH2) and a protein tyrosine phosphatase (PTP) domain. The N-SH2 domain is critical for SHP2 regulation. In the auto-inhibited state, it binds to the PTP domain and blocks the active site, but phosphoprotein engagement destabilizes the N-SH2/PTP domain interaction, thereby exposing the active site. Many disease mutations in SHP2 are at the N-SH2/PTP interface, and they hyperactivate SHP2 by disrupting auto-inhibitory interactions. The activating E139D mutation represents an exception to this mechanism, as it resides in the C-SH2 domain and makes minimal interactions in auto-inhibited and active state crystal structures. In this study, using AlphaFold2 modeling and molecular dynamics simulations, we identify an alternative active conformation of SHP2, in which Glu139 interacts with Arg4 and Arg5 on the N-SH2 domain to stabilize a novel N-SH2/C-SH2 interface. Using double mutant cycles, we show that this active state is further stabilized by the E139D mutation and is dependent on Arg5. Finally, we demonstrate that the E139D mutation enforces an active conformation with distinct phosphoproteins binding preferences from canonical hyperactive mutants. Thus, our study reveals a novel mechanism for SHP2 dysregulation.

## Introduction

SHP2 is a protein tyrosine phosphatase with diverse and critical roles in cell signaling and human physiology^1-3^. The importance of SHP2 function is further underscored through the many disease-associated missense mutations that have been identified in *PTPN11*, the gene encoding SHP2. Mutations in SHP2 are primarily known for their role in the developmental disorders Noonan Syndrome and Noonan Syndrome with Multiple Lentigines^4,5^. However, SHP2 mutations can also drive cancer: 40% of juvenile myelomonocytic leukemia (JMML) patients have SHP2 mutations^6^, and less frequent mutations are also found in acute myeloid leukemia (AML), acute lymphoid leukemia (ALL), and solid cancers^7-9^.

**Figure 1.**
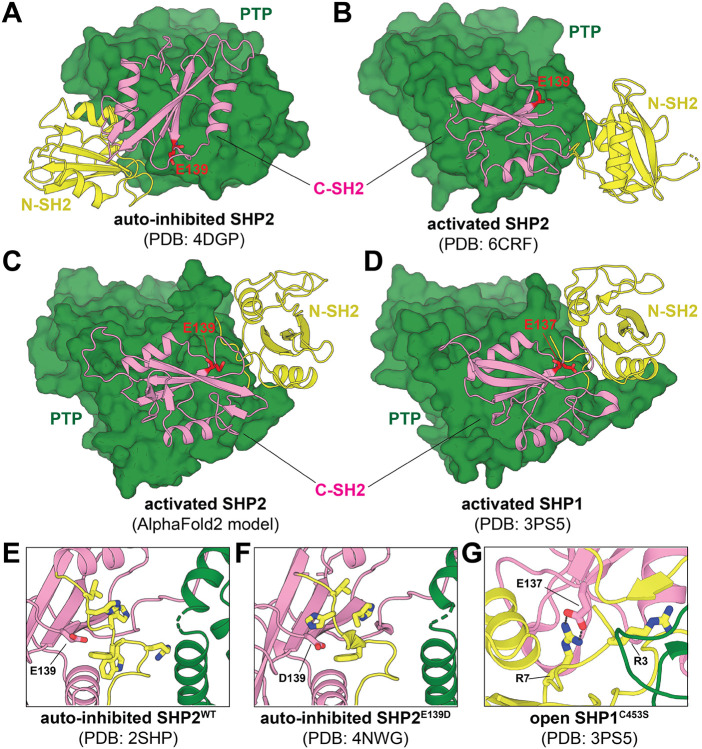
Auto-inhibited and open conformations of SHP2. (**A**) SHP2 exists in an auto-inhibited conformation maintained by auto-inhibitory interactions between the N-SH2 (*yellow*) and PTP domain (*green*) (PDB code 4DGP). (**B**) SHP2^E76K^ adopts an open conformation which is catalytically active. SH2 domains are rotated and N-SH2 domain adopts a dramatically different orientation towards the other domains (PDB code 6CRF). (**C**) AlphaFold2 structure of SHP2 appears similar, but not identical to, the known open conformation of SHP2. The N-SH2 domain exists in a different position and orientation. (**D**) Open conformation of SHP1^C453S^ largely resembles the AlphaFold2 SHP2 state and shows similar rotation of SH2 domains away from PTP domain (PDB code 3PS5). (**E**) Glu139 in the auto-inhibited state does not make any interdomain contacts (PDB code 2SHP). (**F**) D139 in auto-inhibited state does not make any interdomain contacts (PDB code 4NWG). (**G**) E137 in open state of SHP1 interacts with R7 (PDB code 3PS5).

The functional consequences of most disease-associated mutations in SHP2 can be readily understood by examining its structure. SHP2 has three globular domains: a catalytic protein tyrosine phosphatase (PTP) domain, and two SH2-domains that regulate SHP2 activation and localization^10^. The N-SH2 and PTP domains make extensive interactions that keep SHP2 in an auto-inhibited conformation in the apo state (Figure 1A). Engagement of the N-SH2 domain by phospholigands disrupts auto-inhibitory interactions between these two domains, which induces a conformational change that makes the catalytic site accessible to substrates^10^. Many mutations in SHP2-related disorders are found at this auto-inhibitory interface, and they hyperactivate SHP2 by disrupting auto-inhibition. Several other mutations are located outside of this interface. Some indirectly disrupt auto-inhibition by destabilizing the N-SH2 core or by altering the positioning of loops that can sterically block the N-SH2/PTP interaction. Yet others dysregulate SHP2 by altering substrate or activator recognition^11-14^. Several mutations outside of the N-SH2/PTP interface activate SHP2 through currently ambiguous mechanisms.

Much of our understanding of the catalytically-active conformation of SHP2 comes from a single crystal structure of the JMML mutant SHP2^E76K^ (Figure 1B, PDB code 6CRF). This mutation severely impedes the auto-inhibitory interactions between the N-SH2 and PTP domains^10,15^. In the published crystal structure, SHP2^E76K^ inhabits an “open conformation”, where the C-SH2 domain pivots on the face of the PTP domain, and the N-SH2 domain is displaced by more than 120° around this C-SH2 pivot axis, leaving the catalytic site exposed and accessible to substrates. In a recent report from our group, we described an AlphaFold2 (AF2) model of wild-type SHP2 in which the C-SH2 domain makes a similar pivot relative to the PTP domain, but the N-SH2 domain is further behind the PTP domain and rotated relative to the C-SH2 domain (Figure 1C)^16^. Some key features of the C-SH2/PTP interface are conserved between this AF2 model and the 6CRF crystallographic active state, and both structures are compatible with the observed mutational sensitivity at the C-SH2/PTP interface seen in our recent deep mutational scanning study^16^. Notably, the SHP2 AF2 model resembles the active conformation of the closely-related phosphatase SHP1, which shows similar alternative positioning and orientation of the SH2 domains relative to the PTP domain and to one another (Figure 1D)^17^. There is growing evidence from small angle X-ray scattering, single molecule fluorescence, and computation that SHP2 can exist in more than one active state^18-21^. Refined structural models of the various states in this ensemble may shed light on mutational effects that cannot currently be explained by existing crystal structures.

SHP2^E139D^ is a pathogenic mutation that exists in both Noonan Syndrome and JMML and has been shown to increase in basal catalytic activity^5,11,13,22^. Interestingly, Glu139 is not located at the auto-inhibitory interface but lies in the C-SH2 domain, and its mechanism of dysregulation remains unknown. Based on the previously determined auto-inhibited structures of SHP2^WT^, there is no obvious role for Glu139 (Figure 1A,E)^23^, and this residue was not resolved in the open structure of SHP2^E76K^ (Figure 1B)^15^. Moreover, no acquired role was found for Asp139 in a crystal structure of SHP2^E139D^, which is in the auto-inhibited state (Figure 1F)^24^. Interestingly, the analogous residue in SHP1, Glu137, engages with Arg7 on the N-SH2 domain in the active state SHP1 structure (Figure 1G)^17^. These observations raise the possibility that the E139D exerts its effects not by destabilizing auto-inhibition but instead by stabilizing an active state of SHP2. This is supported by the finding that E139D is the only substitution at this residue that is hyperactivating in our previously reported deep mutational scan of SHP2 (Supplementary Figure 1)^16^.

Here, we describe the biochemical characterization of key residues in an interaction network involving Glu139/Asp139 that were observed in molecular dynamics (MD) simulations of the AF2-predicted open state. We demonstrate that the hyperactivating effect of SHP2^E139D^ is likely mediated by stabilizing interactions between Asp139 and Arg5 in this non-crystallographic open state. We also provide evidence that E139D stabilizes a specific open conformational state with altered phosphoprotein binding preference compared to other SHP2 variants. Our results shed light on a potential molecular mechanism underlying the pathogenicity of the E139D mutation and further support the conformational ensemble model for active SHP2.

## Results

### Glu139/Asp139 interacts with N-terminal Arg residues in an alternative SHP2 open conformation

**Figure 2.**
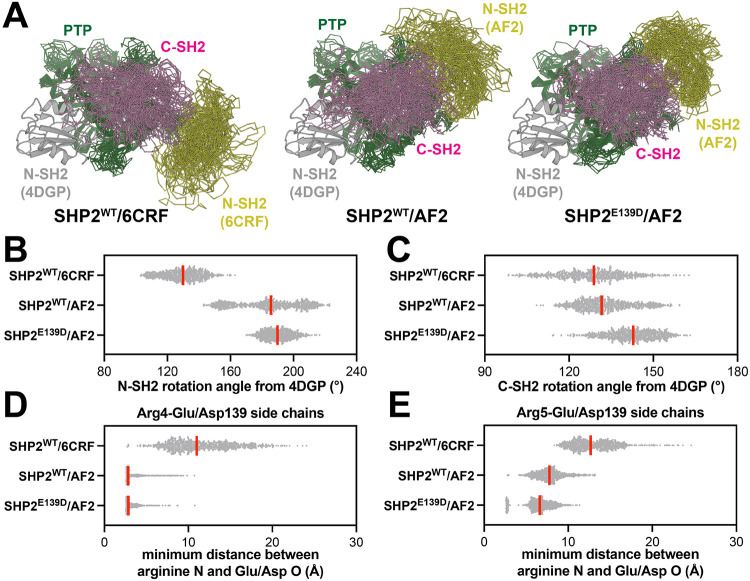
A non-crystallographic open conformation of SHP2 is stabilized by Glu139/Asp139 electrostatic interactions in molecular dynamics simulations. (**A**) Representative sampling of structures from MD simulation trajectories of SHP2^WT^/6CRF (*left*), SHP2^WT^/AF2 (*middle*), and SHP2^E139D^/AF2 (*right*). Yellow, pink, and green represent N-SH2, C-SH2, and PTP domains, respectively. N-SH2 domain positioning in the closed conformation (PDB code 4DGP) is shown in gray as a reference, and all states are aligned over just the PTP domain residues. (**B**) Distribution of N-SH2 rotation angles from the closed conformation (PDB code 4DGP) to the positions seen in the SHP2^WT^/6CRF, SHP2^WT^/AF2, and SHP2^E139D^/AF2 simulations. (**C**) Same as (**B**) but measuring C-SH2 rotation. (**D**) Minimum distance between Arg4 side-chain N atoms and Glu139/Asp139 side-chain O atoms in the SHP2^WT^/6CRF, SHP2^WT^/AF2, and SHP2^E139D^/AF2 simulations. (**E**) Same as (**D**), but for Arg5.

To compare interdomain orientation and dynamics in different open conformations of SHP2, we revisited our recently published triplicate 2.5 [μs MD simulations starting from the open conformation crystal structure (PDB code 6CRF) and the AF2 model, both with the SHP2^WT^ sequence^16^. On the timescale of these simulations, both SHP2^WT^/6CRF and SHP2^WT^/AF2 sample a range of positions and orientations of the N-SH2 domain relative to the C-SH2 and PTP domains (Figure 2A). We quantified this movement by measuring an angle defined by N-SH2 position in the auto-inhibited state (PDB code 4DGP), a pivot point on the C-SH2 domain, and N-SH2 position in the open state simulations (Supplementary Figure 2A). In general, the N-SH2 domain retained a larger rotation angle in the SHP2^WT^/AF2 simulations relative to the SHP2^WT^/6CRF simulations (Figure 2B). The C-SH2 domain itself also rotates between the auto-inhibited and open states, while sitting on the face of the PTP domain (Supplementary Figure 2B). The C-SH2 domain rotation was larger in SHP2^WT^/AF2 simulations when compared to the SHP2^WT^/6CRF simulations (Figure 2C).

As a consequence of these different interdomain orientations, Glu139 was substantially more buried in SHP2^WT^/AF2 simulations when compared with SHP2^WT^/6CRF simulations (Supplementary Figure 3). Another consequence of this change in interdomain dynamics was that Arg4 and Arg5 at the N-terminus were proximal to Glu139 in the SHP2^WT^/AF2 simulations, with the Arg4 guanidinium group persistently ion pairing with the Glu139 carboxylate (Figure 2D,E). By contrast, the two arginine residues are further away from Glu139 in the SHP2^WT^/6CRF simulations (Figure 2D,E). This Glu139-Arg4 interaction is similar to the interaction between Glu137 and Arg7 in the open conformation crystal structure of SHP1 (Figure 1G)^17^. We hypothesize that these electrostatic interactions play a role in stabilizing the SHP1 and SHP2^WT^/AF2 active conformations and are not relevant to the distinct SHP2^WT^/6CRF active conformation.

To evaluate how the E139D mutation might alter interactions at the N-SH2/C-SH2 interface, we conducted triplicate 2.5 [μs MD simulations starting from our original AF2 active state model, but with the Glu139 mutated to Asp. Asp139 remained buried at the N-SH2/C-SH2 interface in these simulations (Supplementary Figure 3). However, when compared to SHP2^WT^/AF2, the SHP2^E139D^/AF2 simulations showed decreased N-SH2 dynamics and a slight increase in the C-SH2 rotation angle (Figure 2A-C). We hypothesize that the shorter side chain of Asp139 stabilizes the further rotated SH2 domains. At the N-SH2/C-SH2 interface, Asp139 interacts with both Arg4 and Arg5, as also seen in the SHP2^WT^/AF2 simulations (Figure 2D,E). However the Asp139-Arg5 interaction frequency increased in the mutant simulations when compared to Glu139-Arg5 in the wild-type simulations. These simulations suggest that Glu139 makes interdomain interactions in the AF2-predicted SHP2 active conformation that are further enhanced with the E139D mutation, and the stabilization of this active state may explain the activating effect seen for this mutation.

### The activating role of Glu139/Asp139 is dependent on Arg4 and Arg5

**Figure 3.**
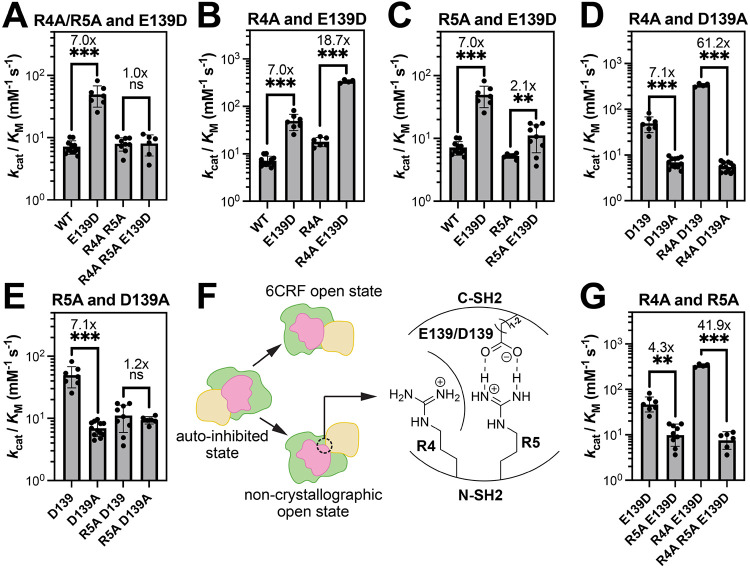
Coupling between Arg4, Arg5 and Glu139/Asp139. (**A**) The activating effect of the mutation E139D is dependent on the presence of Arg4/Arg5. (**B**) The R4A mutation enhances the activating effect of E139D. (**C**) The R5A mutation decreases the activating effect of E139D. (**D**) The inactivating effect of the D139A mutation is amplified in an R4A background. (**E**) The inactivating effect of the D139A mutation is lost in an R5A background. (**F**) Cooperation of Arg4, Arg5 and Glu139/Asp139 in the non-crystallographic open conformation: Arg5 interacts with Glu139/Asp139 to stabilize the non-crystallographic open conformation, while Arg4 inhibits this stabilization. (**G**) The inactivating effect of the R5A mutation in an E139D background is significantly enhanced by the R4A mutation. In all panels, statistical significance was assessed by Welch’s two-tailed t test. ns = not significant, * denotes p < 0.05, ** denotes p < 0.01, *** denotes p < 0.001.

To evaluate the functional effects of Arg4/Arg5 hydrogen bonding to Glu139/Asp139, we experimentally characterized SHP2 single mutations at these residues, as well as a series of double and triple mutants (Supplementary Table 1). We first compared the catalytic efficiencies (k_cat_/KM values) of SHP2^WT^, SHP2^E139D^, SHP2^R4A+R5A^, and SHP2^R4A+R5A+E139D^ using 6,8-difluoro-4-methylumbelliferyl phosphate (DiFMUP), a commonly used fluorogenic phosphatase substrate. As expected, the E139D mutation increases SHP2 basal activity 7-fold. However, in the background of the R4A+R5A double mutation, E139D no longer altered SHP2 activity (Figure 3A). These results demonstrate that the activating effect of Asp139 is dependent on the presence of Arg4 and/or Arg5. To dissect the roles of Arg4 and Arg5, we performed additional mutant cycles analyzing R4A and R5A separately with E139D. Interestingly, we found that the R4A mutation itself mildly increased SHP2 activity (Supplementary Figure 4A), and this removal of the Arg4 side chain amplified the activating effect of E139D from a 7-fold to 18.7-fold enhancement in activity (Figure 3B). On the other hand, the R5A mutation alone had a modest negative effect on SHP2 activity (Supplementary Figure 4B), and it significantly weakened the activating effect of E139D from 7-fold to 2.1-fold (Figure 3C and Supplementary Figure 4B). Together, these data suggest that Arg5 enables SHP2 activation by E139D, while Arg4 inhibits this effect.

Our AF2 model and MD simulations not only suggest how the E139D mutation can stabilize an active SHP2 conformation, but they also suggest that Glu139 might be able to engage Arg4 and Arg5 in wild-type context, albeit to a lesser extent than Asp139. To investigate the interaction between Arg4, Arg5, and Glu139 in SHP2^WT^, we further performed mutant cycle analyses measuring activity levels of SHP2^R4A^, SHP2^R5A^, SHP2^E139A^, and the corresponding double mutants. The E139A mutation did not have a measurable impact on catalytic activity on its own, consistent with our deep mutational scanning data (Supplementary Figure 1 and Supplementary Figure 5A)^16^. Thus, the ability of Glu139 to stabilize an active state in SHP2^WT^ is negligible. When the E139A mutation was made in an R4A background, however, we observed a 3.2-fold reduction in activity (Supplementary Figure 5A). This suggests that Arg4 may be repressing the ability of Glu139 to stabilize the active state, and so the effect of E139A is only detected when Arg4 is removed. On the other hand, the R5A mutation only causes a very modest 1.8-fold enhancement in the effect of the E139A mutation (Supplementary Figure 5B). These data indicate that in wild-type SHP2, Glu139 may not effectively engage Arg5, but any role Glu139 it has in stabilizing the active state is suppressed by the presence of Arg4.

Finally, we compared constructs with E139D to those with E139A to analyze the impact of removing an Asp side chain rather than Glu side chain. In this analysis, we see an amplification of these effects seen with E139A: The “D139A” mutation (i.e. comparison of E139D to E139A) inactivated SHP2 7.1-fold, and this becomes a 61.2-fold reduction in activity in an R4A background (Figure 3D and Supplementary Figure 5C). By contrast, the negative effect of the “D139A” mutation is fully lost in an R5A background (Figure 3E and Supplementary Figure 5D). Together, our data clearly demonstrate that there is an interplay between Arg4, Arg5, and Glu/Asp139 to facilitate SHP2 activity, but that the roles of Arg4 and Arg5 are opposing. We hypothesize that Arg5 directly interacts with Glu139 in SHP2^WT^ and more strongly with Asp139 in SHP2^E139D^ through hydrogen bonding or ionic interactions to activate SHP2.

Our MD simulations suggests that activation by E139D likely results from the stabilization of a non-crystallographic SHP2 open conformation. Although the simulations predict that Arg4, not Arg5, is the major stabilizer of the AlphaFold2-modeled open conformation, our biochemical data show the opposite. Nonetheless, the strong coupling between Arg4, Arg5, and Glu/Asp139, suggest that these residues are in close proximity. Thus, our structural model likely approximates the true active state of SHP2^E139D^ and is likely an accessible conformational state for SHP2^WT^. We hypothesize that a Glu/Asp139-Arg5 ion pair is a key stabilizing interaction in this conformational state, and Arg4 might competitively disrupt this interaction, perhaps through steric or electrostatic repulsion of Arg5 (Figure 3F). Indeed, the detrimental effects of removing the Arg5 side chain in an E139D background are amplified when the Arg4 side chain is removed, showing that these two residues are thermodynamically coupled (Figure 3G). However, we note that Arg4 interacts with Glu258 in the auto-inhibited crystal structure^10^ and with Glu485 in the 6CRF crystal structure^15^. Thus, a simple interpretation of R4A mutational effects is convoluted by the role of Arg4 in multiple conformational states.

### The activating E76K and E139D mutations have distinct effects on SHP2 structure

**Figure 4.**
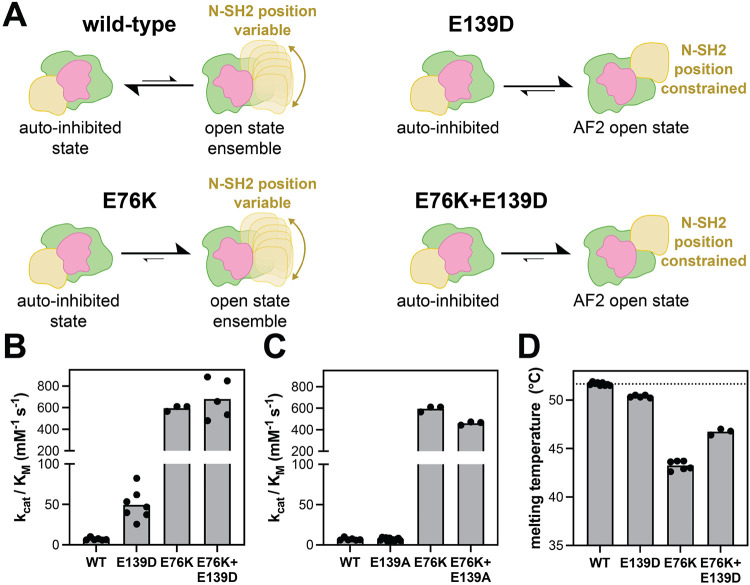
Divergent effects of E76K and E139D mutations on SHP2 activity and stability. (**A**) Cartoon schematic of the mutational effects of E76K and E139D on the SHP2 open-close equilibrium and on SHP2 structure, particularly N-SH2 positioning. (**B**) Basal catalytic activity measurements show comparable activity for SHP2^E76K^ and SHP2^E76K+E139D^. (**C**) Basal catalytic activity measurements show a slight reduction in activity for SHP2^E76K+E139A^ mutant compared to SHP2^E76K^. (**D**) Differential scanning fluorimetry measurements suggests that the E76K mutation destabilizes the auto-inhibited state, whereas the E139D mutation stabilizes an open state.

The E76K mutation serves as the canonical example of an activating SHP2 mutation at the N-SH2/PTP interface. SHP2^E76K^ is one of the most active SHP2 mutants, it can enhance basal activity against DiFMUP over 100-fold, and it cannot be further activated by phospho-peptide ligands, suggesting that it does not appreciably occupy the auto-inhibited state^15,16^. Disruption of the auto-inhibited conformation by this mutation has been extensively characterized, and indeed, SHP2^E76K^ provided the first experimentally determined crystal structure of an open SHP2 conformation^15^. However, accumulating evidence suggests that this crystallographic structure represents just one of multiple open conformations that SHP2 can access. Early crystallization attempts with full-length SHP2^E76K^ failed, attributed to the flexibility of the N-SH2 domain^19^. Subsequent experimental work confirmed alternative N-SH2 positions, supporting the existence of open conformations distinct from that captured in the SHP2^E76K^ crystal structure^19^. More recent studies indicate that the active state of SHP2 is better described as a dynamic, structurally heterogeneous ensemble rather than a single static conformation^21^. Consistent with this view, other activating mutations in SHP2 have also been shown to populate open states, and studies of SHP2^E76A^ revealed a broad distribution of interdomain distances, again pointing to the presence of multiple accessible open conformations^25^. Thus, while the E76K mutation relieves auto-inhibition and is therefore largely incompatible with the closed conformation, it is able to access a wide range of open states (Figure 4A).

By contrast, based on our AlphaFold2 model and biochemical results, the E139D mutation appears to stabilize a specific position and orientation of the N-SH2 domain in the open state (Figure 4A). However, based on the fact that the E139D mutation only increases basal activity 7-fold, and this mutation does not impact the auto-inhibitory interface, we hypothesize that SHP2^E139D^ still rests partly in the auto-inhibited state, unlike SHP2^E76K^. To further characterize the differences between these two mutations, we made an E76K+E139D double mutant. Whereas the E76K mutation alone should fully destabilize the auto-inhibited state to yield an ensemble of active states, addition of the E139D mutation might “trap” SHP2 in a specific open state (Figure 4A). First, we measured catalytic efficiencies for both single mutants and the double mutant (Figure 4B). While both single mutants increase catalytic activity, as expected, SHP2^E76K^ is demonstrably much more active with the DiFMUP substrate than SHP2^E139D^. The SHP2^E76K+E139D^ double mutant was comparable to SHP2^E76K^ in terms of activity (Figure 4B). This suggests that the conformational state stabilized by E139D can achieve maximal SHP2 catalytic activity. As a control, we also made the E76K+E139A double mutant. We found that the activity of SHP2^E76K+E139A^ was slightly but significantly lower than that of SHP2^E76K^ alone (p-value < 0.01) (Figure 4C). Taken together, our data suggests that Glu139 stabilizes an active conformation, Asp139 enhances this stabilizing interaction, whereas this is lost in Ala139.

Next, we performed differential scanning fluorimetry to measure the thermal stability and conformation of SHP2 (Supplementary Table 2). Previous work has shown that melting temperature (T_m_) can report on SHP2 conformation^16,26,27^. Specifically, mutants in which the auto-inhibited state is destabilized (e.g. SHP2^E76K^) have lower T_m_ values than SHP2^WT^, most likely due to fewer interdomain contacts. By extension, given that SHP2^E139D^ stabilizes a specific open conformation with new interdomain contacts, we hypothesized that it may have a higher T_m_ value than SHP2^E76K^. Thus, we measured the melting temperatures for both single mutants and the SHP2^E76K+E139D^ double mutant (Figure 4D). Consistent with previous work, we observed a large decrease in melting temperature for SHP2^E76K^ when compared to SHP2^WT 11,16,26^. By comparison, SHP2^E139D^ only showed a slight decrease in T_m_ relative to SHP2^WT^. Notably, the SHP2^E76K+E139D^ double mutant had a higher T_m_ than SHP2^E76K^, despite the fact that both variants should not appreciably adopt the auto-inhibited state. This is consistent with the idea that the E139D mutation enhances specific interdomain contacts in an active/open conformation, whereas E76K merely destabilizes the auto-inhibited state (Figure 4A)

### The E139D mutation alters the phosphoprotein binding preferences of SHP2

**Figure 5.**
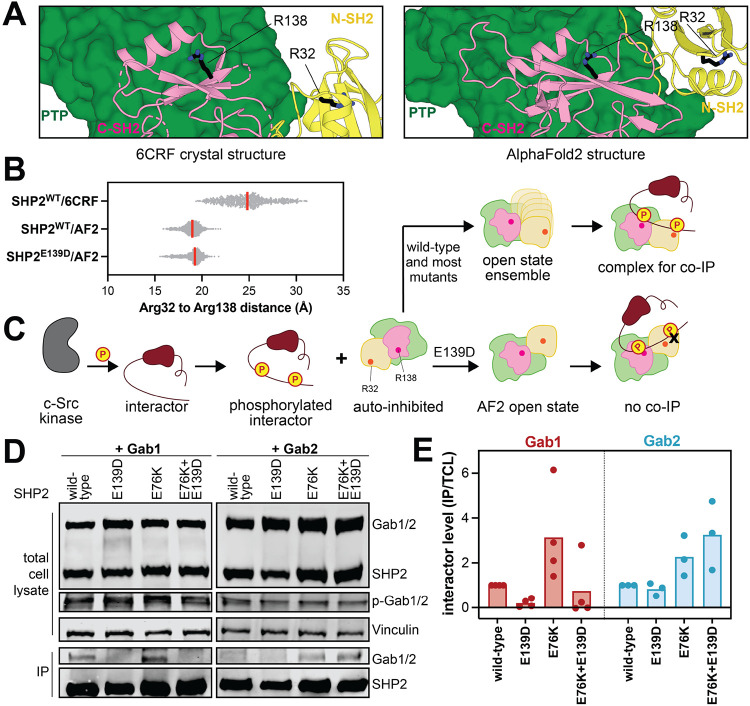
The E139D mutation alters the scope of SHP2-phosphoprotein interactions. (**A**) Positions of Arg32 and Arg138 in 6CRF and AF2 structures. (**B**) Distances (Å) between Arg32 and Arg138 Cα atoms in SHP2^WT^/6CRF, SHP2^WT^/AF2, and SHP2^E139D^/AF2 simulations. (**C**) Schematic depicting how the E139D-stabilized open state might impact phosphoprotein binding. (**D**) Representative western blots showing altered co-immunopurification trends for Gab1 and Gab2 by the E139D mutation. (**E**) Quantification of SHP2 co-immunopurification results for Gab1 and Gab2 based on 3 biological replicates for each interacting protein. Interactor levels are normalized to Gab1/Gab2 protein levels in the total cell lysate (TCL), then further normalized to Gab1/Gab2 levels immunopurified in the wild-type sample.

SHP2 is known to tightly bind bis-phosphorylate peptides and proteins through simultaneous engagement of the N-SH2 and C-SH2 domains with nearby phosphosites^22,28,29^. Indeed, bis-phosphorylated molecules are some of the most potent activators of SHP2 and play a key role in coordinating SHP2 signaling^18,30^. Given that the E139D mutation appears to stabilize a specific open state, we reasoned that this might narrow the range of possible SH2 orientations relative to one another, thereby altering binding preferences for bis-phosphorylated proteins. To test this, we first analyzed MD trajectories. We measured the dihedral angles between two planes defined by the center of mass of either SH2 domain (Cα of L43 and L149) and the Cα of Arg111 and Arg220 on the linkers (Supplementary Figure 6A). This dihedral angle is consistently sharper in the SHP2^WT/E139D^/AF2 simulations when compared to the SHP2^WT^/6CRF simulations, correlating with the further rotation of the N-SH2 domain relative to the C-SH2 domain (Supplementary Figure 6B). This repositioning of SH2 domains can also alter the orientation of the phosphotyrosine binding residues in the N- and C-SH2 domains. We measured the distance between the Cζ of the two phosphotyrosine binding arginine residues in the SH2 domains, Arg32 and Arg138, in both the 6CRF and the AF2 simulations (Figure 5A). Our data shows that the arginine residues in the N- and C-SH2 domain are in closer proximity in the AF2 model than in the SHP2^E76K^ crystal structure, as well as in the AF2- and 6CRF-derived simulations (Figure 5B). Moreover, the AF2 simulations show a tighter range of distances between Arg32 and Arg138.

To examine whether the spatial constraints suggested by the AF2 model and our MD simulations could impact SHP2 interactions, we assessed the ability of different SHP2 variants to bind to two different bis-phosphorylated proteins that are known SHP2 interactors, Gab1 and Gab2. We reasoned that, if SHP2^E139D^ merely adopts any more open conformation, this would enhance the pull-down of the interacting phosphoprotein, as we have previously shown for SHP2^E76K^ ^11^. However, if SHP2^E139D^ adopts a conformation incompatible with the distance between the phosphosites on the interacting protein, we would expect to see a reduction of co-immunopurification compared to SHP2^WT^ or SHP2^E76K^ (Figure 5C). Thus, we co-expressed SHP2 and Gab1 or Gab2 in HEK 293 cells. A constitutively active variant of c-Src was also co-expressed to ensure Gab1/Gab2 phosphorylation. SHP2 was immuno-purified, and levels of co-immunopurified Gab1/Gab2 were assessed by western blot. Interestingly, compared to SHP2^WT^ we saw a reduction of Gab1 binding for SHP2^E139D^, whereas Gab2 binding was similar between the two SHP2 variants (Figure 5D,E). By contrast, SHP2^E76K^ showed increased immunopurification for both Gab1 and Gab2, as expected. Finally, we also made a double mutant of SHP2^E76K+E139D^ to eliminate any effect of the auto-inhibited state and instead only consider the effect of E139D in stabilizing specific open conformation. Addition of the E139D mutation on top of the E76K mutation reduced binding to Gab1 when compared to SHP2^E76K^, consistent with the conformationally constraining role of the E139D mutation. By contrast, the SHP2^E76K+E139D^ double mutant had similar or enhanced immunopurification of Gab2 relative to SHP2^E76K^, suggesting that the E139D mutation stabilizes a state that is compatible with Gab2 binding (Figure 5D,E). These results support the hypothesis that the E139D mutation stabilizes a specific open conformation that can alter SHP2 ligand binding compatibility.

One alternative explanation for our co-immunopurification results is that the E139D mutation alters the intrinsic binding properties of the C-SH2 domain. Indeed, this residue is directly adjacent to R138, which is essential for phosphoprotein binding^11,30^. However, we previously showed that the E139D mutation does not appreciably impact binding affinity or specificity against array of phosphopeptides, including those derived from the Gab1 and Gab2 phosphosites^11^. Thus, it is more likely that the E139D mutation alters binding preferences through a change in interdomain orientation and dynamics. In support of this model, we analyzed our previously reported proximity-labeling proteomics data for SHP2^WT^, SHP2^E76K^, and SHP2^E139D^ ^31^. These three variants share a core interactome but also show differences in proximity labeling for a number of proteins (Supplementary Figure 7A). This includes a SHP2^E139D^-specific lack of significant labeling of Gab1 and an enhancement in significant labeling of MPZL1, another disease-relevant SHP2 interactor^32^, over the negative control (Supplementary Figure 7B). Notably, another study that used the SHP2 tandem SH2 domains for immunopurification and phosphoproteomics also reported different interactomes for the wild-type and E139D tandem SH2 domains^33^. Collectively, our results show that the E139D mutation can impact not only SHP2 basal activity, but also SHP2 protein-protein interaction specificity, which could contribute to the pathogenicity of this variant.

## Discussion

The first structure of SHP2 in a catalytically-competent state, determined using the E76K mutant, was a major milestone for the SHP2 field^15^. Since then, it has become evident that SHP2 can access multiple open conformations and that the SHP2^E76K^ crystal structure represents just one of the possible states^19-21,25^. Using AlphaFold2, we obtained an open SHP2 conformation distinct from the SHP2^E76K^ structure — one that more closely resembles the open conformation previously described for SHP1^17^. Notably, in the open conformation crystal structure of SHP1^C453S^ residue Glu137 (the equivalent of SHP2 Glu139) forms interactions with an arginine residue near the N-terminus. These observations led us to probe whether SHP2 can access a comparable structural state, if an interaction between Glu139 and similar arginine residues in SHP2 might exist, and whether such an interaction could help explain the pathogenic effect of the E139D mutation.

Closer analysis of our previously published molecular dynamics simulations of the AlphaFold2 model revealed a role for Glu139 in stabilizing this novel active state through electrostatic interactions with Arg4 and Arg5^16^. Through biochemical measurements in this study, we have now shown that the activating effect of the E139D mutation is lost in the background of an R5A mutation, suggesting that Arg5 favors SHP2 activity through energetical coupling with Glu139/Asp139. This observation cannot be explained through current crystallographic states of SHP2^10,15,23^, and it and supports the AlphaFold2 model as one plausible alternative open conformation. This role for Glu139 in stabilizing new interdomain interactions in an active state provides insights into how E139D could potentially affect SHP2 stability and function. We hypothesize that the shorter side chain of aspartate may enable a more energetically favorable and stable interaction with Arg5 and enhance N-SH2/C-SH2 interactions, thereby more effectively stabilizing the AF2-modeled active conformation. This would explain why only the E139D mutation, and no other substitutions at this position, is activating.

Unlike the clear roles for Glu/Asp139 and Arg5, which make interactions almost exclusively in the AF2-modeled conformation, the role of Arg4 in controlling SHP2 structure appears multi-faceted. In the auto-inhibited state, Arg4 forms a salt bridge with Glu258^10^. By contrast, in the SHP2^E76K^ crystal structure, Arg4 forms a salt bridge with Asp485 in the PTP domain that may be stabilizing. In our experiments, loss of Arg4 (R4A mutation) increased basal activity of SHP2. However, this activating effect was reduced if there was a concurrent loss of Arg5 (R4A R5A mutation). Moreover, the loss of Arg4 was catalytically-neutral in the context of the Ala139, suggesting that presence of an acidic residue on position 139 is critical for the activating effect of R4A. We speculate that while Arg4 can stabilize both the auto-inhibited and some of the active conformations, its presence might hinder Arg5 from optimally engage with Glu139, so that loss of Arg4 could indirectly favor activation through Arg5.

According to our simulations, one consequence of the stabilization of the AF2-modeled state is a narrower range of distances between the two phosphotyrosine-binding pockets in the SHP2 tandem SH2 domains. Binding experiments with two known bis-phosphorylated SHP2 interactors, Gab1 and Gab2, show that the active-state conformational constraint placed by the E139D mutation biases its binding specificity. This is in stark contrast to mutations like E76k, which activate SHP2 by destabilizing the active state but do not enforce a specific active state. An important consequence of this difference is that E139D may bind to and be activated by a different spectrum of upstream phosphoproteins compared to SHP2^WT^ and other SHP2 mutants. The downstream signaling outcomes of this interactome rewiring will require further investigation.

In summary, our results suggest that Glu139 stabilizes a novel active state of SHP2 through interactions with Arg5, and that substitution to Asp139 at this position strengthens this interaction, providing a mechanistic explanation for the activating effect of E139D. More broadly, this work underscores the importance of considering protein conformational dynamics and uncharacterized protein states when interpreting the functional consequences of disease-associated mutations.

## Supplementary Material

Supplement 1 Supp. Figure 1. Unique activating effect of the E139D mutation.
Supp. Figure 2. Measurements of SH2 domain position and rotation.
Supp. Figure 3. Solvent accessible surface area of Glu/Asp139 in MD simulations.
Supp. Figure 4. Mutant cycle analyses with R4A, R5A, and E139D.
Supp. Figure 5. Mutant cycle analyses with R4A, R5A, and E/D139A.
Supp. Figure 6. Interdomain dihedral angle analysis showing SH2 domain repositioning.
Supp. Figure 7. Comparison of proximity-labeling hits for WT, E76K, and E139D.

Supplement 2 Supp. Table 1. Catalytic efficiencies of all SHP2 variants used in this study.

Supplement 3 Supp. Table 2. Melting temperatures of all SHP2 variants used in this study.

## References

[1] Scheiter A.; Lu L.-C.; Gao L. H.; Feng G.-S. Complex Roles of PTPN11/SHP2 in Carcinogenesis and Prospect of Targeting SHP2 in Cancer Therapy. Annual Review of Cancer Biology 2024, 8 (1), 15–33. 10.1146/annurev-cancerbio-062722-013740. PMC11824402 39959686

[2] Lauriol J.; Jaffré F.; Kontaridis M. I. The Role of the Protein Tyrosine Phosphatase SHP2 in Cardiac Development and Disease. Seminars in Cell & Developmental Biology 2015, 37, 73–81. 10.1016/j.semcdb.2014.09.013. 25256404 PMC4339543

[3] Salmond R. J.; Alexander D. R. SHP2 Forecast for the Immune System: Fog Gradually Clearing. Trends in Immunology 2006, 27 (3), 154–160. 10.1016/j.it.2006.01.007. 16458607

[4] Edouard T.; Combier J.-P.; Nédélec A.; Bel-Vialar S.; Métrich M.; Conte-Auriol F.; Lyonnet S.; Parfait B.; Tauber M.; Salles J.-P.; Lezoualc’h F.; Yart A.; Raynal P. Functional Effects of PTPN11 (SHP2) Mutations Causing LEOPARD Syndrome on Epidermal Growth Factor-Induced Phosphoinositide 3-Kinase/AKT/Glycogen Synthase Kinase 3beta Signaling. Molecular and Cellular Biology 2010, 30 (10), 2498–2507. 10.1128/MCB.00646-09. 20308328 PMC2863708

[5] Tartaglia M.; Martinelli S.; Stella L.; Bocchinfuso G.; Flex E.; Cordeddu V.; Zampino G.; van der Burgt I.; Palleschi A.; Petrucci T. C.; Sorcini M.; Schoch C.; Foa R.; Emanuel P. D.; Gelb B. D. Diversity and Functional Consequences of Germline and Somatic PTPN11 Mutations in Human Disease. American Journal of Human Genetics 2006, 78 (2), 279–290. 10.1086/499925. 16358218 PMC1380235

[6] Gupta A. K.; Meena J. P.; Chopra A.; Tanwar P.; Seth R. Juvenile Myelomonocytic Leukemia-A Comprehensive Review and Recent Advances in Management. American journal of blood research 2021, 11 (1), 1–21. 33796386 PMC8010610

[7] Fobare S.; Kohlschmidt J.; Ozer H. G.; Mrózek K.; Nicolet D.; Mims A. S.; Garzon R.; Blachly J. S.; Orwick S.; Carroll A. J.; Stone R. M.; Wang E. S.; Kolitz J. E.; Powell B. L.; Oakes C. C.; Eisfeld A.-K.; Hertlein E.; Byrd J. C. Molecular, Clinical, and Prognostic Implications of PTPN11 Mutations in Acute Myeloid Leukemia. Blood Advances 2022, 6 (5), 1371–1380. 10.1182/bloodadvances.2021006242. 34847232 PMC8905707

[8] Estey E. H. Acute Myeloid Leukemia: 2019 Update on Risk-Stratification and Management. Am J Hematol 2018, 93 (10), 1267–1291. 10.1002/ajh.25214. 30328165

[9] Bentires-Alj M.; Paez J. G.; David F. S.; Keilhack H.; Halmos B.; Naoki K.; Maris J. M.; Richardson A.; Bardelli A.; Sugarbaker D. J.; Richards W. G.; Du J.; Girard L.; Minna J. D.; Loh M. L.; Fisher D. E.; Velculescu V. E.; Vogelstein B.; Meyerson M.; Sellers W. R.; Neel B. G. Activating Mutations of the Noonan Syndrome-Associated SHP2/PTPN11 Gene in Human Solid Tumors and Adult Acute Myelogenous Leukemia. Cancer Research 2004, 64 (24), 8816–8820. 10.1158/0008-5472.CAN-04-1923. 15604238

[10] Hof P.; Pluskey S.; Dhe-Paganon S.; Eck M. J.; Shoelson S. E. Crystal Structure of the Tyrosine Phosphatase SHP-2. Cell 1998, 92 (4), 441–450. 10.1016/s0092-8674(00)80938-1. 9491886

[11] Van Vlimmeren A. E.; Voleti R.; Chartier C. A.; Jiang Z.; Karandur D.; Humphries P. A.; Lo W.-L.; Shah N. H. The Pathogenic T42A Mutation in SHP2 Rewires the Interaction Specificity of Its N-Terminal Regulatory Domain. Proc Natl Acad Sci U S A 2024, 121 (30), e2407159121. 10.1073/pnas.2407159121. 39012820 PMC11287265

[12] Toto A.; Malagrinò F.; Visconti L.; Troilo F.; Gianni S. Unveiling the Molecular Basis of the Noonan Syndrome-Causing Mutation T42A of SHP2. International Journal of Molecular Sciences 2020, 21 (2). 10.3390/ijms21020461. PMC7013464 31936901

[13] Martinelli S.; Torreri P.; Tinti M.; Stella L.; Bocchinfuso G.; Flex E.; Grottesi A.; Ceccarini M.; Palleschi A.; Cesareni G.; Castagnoli L.; Petrucci T. C.; Gelb B. D.; Tartaglia M. Diverse Driving Forces Underlie the Invariant Occurrence of the T42A, E139D, I282V and T468M SHP2 Amino Acid Substitutions Causing Noonan and LEOPARD Syndromes. Human Molecular Genetics 2008, 17 (13), 2018–2029. 10.1093/hmg/ddn099. 18372317 PMC2900904

[14] Zhang R.-Y.; Yu Z.-H.; Chen L.; Walls C. D.; Zhang S.; Wu L.; Zhang Z.-Y. Mechanistic Insights Explain the Transforming Potential of the T507K Substitution in the Protein-Tyrosine Phosphatase SHP2. The Journal of Biological Chemistry 2020, 295 (18), 6187–6201. 10.1074/jbc.RA119.010274. 32188694 PMC7196634

[15] LaRochelle J. R.; Fodor M.; Vemulapalli V.; Mohseni M.; Wang P.; Stams T.; LaMarche M. J.; Chopra R.; Acker M. G.; Blacklow S. C. Structural Reorganization of SHP2 by Oncogenic Mutations and Implications for Oncoprotein Resistance to Allosteric Inhibition. Nat Commun 2018, 9 (1), 4508. 10.1038/s41467-018-06823-9. 30375388 PMC6207684

[16] Jiang Z.; Van Vlimmeren A. E.; Karandur D.; Semmelman A.; Shah N. H. Deep Mutational Scanning of the Multi-Domain Phosphatase SHP2 Reveals Mechanisms of Regulation and Pathogenicity. Nat Commun 2025, 16, 5464. 10.1038/s41467-025-60641-4. 40595497 PMC12216643

[17] Wang W.; Liu L.; Song X.; Mo Y.; Komma C.; Bellamy H. D.; Zhao Z. J.; Zhou G. W. Crystal Structure of Human Protein Tyrosine Phosphatase SHP-1 in the Open Conformation. J Cell Biochem 2011, 112 (8), 2062–2071. 10.1002/jcb.23125. 21465528 PMC3135737

[18] Hayashi T.; Senda M.; Suzuki N.; Nishikawa H.; Ben C.; Tang C.; Nagase L.; Inoue K.; Senda T.; Hatakeyama M. Differential Mechanisms for SHP2 Binding and Activation Are Exploited by Geographically Distinct Helicobacter Pylori CagA Oncoproteins. Cell reports 2017, 20 (12), 2876–2890. 10.1016/j.celrep.2017.08.080. 28930683

[19] Pádua R. A. P.; Sun Y.; Marko I.; Pitsawong W.; Stiller J. B.; Otten R.; Kern D. Mechanism of Activating Mutations and Allosteric Drug Inhibition of the Phosphatase SHP2. Nat Commun 2018, 9 (1), 4507. 10.1038/s41467-018-06814-w. 30375376 PMC6207724

[20] Tao Y.; Xie J.; Zhong Q.; Wang Y.; Zhang S.; Luo F.; Wen F.; Xie J.; Zhao J.; Sun X.; Long H.; Ma J.; Zhang Q.; Long J.; Fang X.; Lu Y.; Li D.; Li M.; Zhu J.; Sun B.; Li G.; Diao J.; Liu C. A Novel Partially Open State of SHP2 Points to a “Multiple Gear” Regulation Mechanism. The Journal of Biological Chemistry 2021, 296, 100538. 10.1016/j.jbc.2021.100538. 33722610 PMC8054191

[21] Anselmi M.; Hub J. S. Atomistic Ensemble of Active SHP2 Phosphatase. Commun Biol 2023, 6 (1), 1289. 10.1038/s42003-023-05682-5. 38129686 PMC10739809

[22] Keilhack H.; David F. S.; McGregor M.; Cantley L. C.; Neel B. G. Diverse Biochemical Properties of Shp2 Mutants. Implications for Disease Phenotypes. The Journal of Biological Chemistry 2005, 280 (35), 30984–30993. 10.1074/jbc.M504699200. 15987685

[23] Yu Z.-H.; Xu J.; Walls C. D.; Chen L.; Zhang S.; Zhang R.; Wu L.; Wang L.; Liu S.; Zhang Z.-Y. Structural and Mechanistic Insights into LEOPARD Syndrome-Associated SHP2 Mutations. The Journal of Biological Chemistry 2013, 288 (15), 10472–10482. 10.1074/jbc.M113.450023. 23457302 PMC3624429

[24] Qiu W.; Lin A.; Hutchinson A.; Romanov V.; Ruzanov M.; Thompson C.; Lam K.; Kisselman G.; Battalie K.; Chirgadze N. Y. Crystal Structure of the Tyrosine Phosphatase SHP-2 with E139D Mutation: 4nwg, 2014. 10.2210/pdb4nwg/pdb.

[25] LaRochelle J. R.; Fodor M.; Xu X.; Durzynska I.; Fan L.; Stams T.; Chan H. M.; LaMarche M. J.; Chopra R.; Wang P.; Fortin P. D.; Acker M. G.; Blacklow S. C. Structural and Functional Consequences of Three Cancer-Associated Mutations of the Oncogenic Phosphatase SHP2. Biochemistry 2016, 55 (15), 2269–2277. 10.1021/acs.biochem.5b01287. 27030275 PMC4900891

[26] Serbina A.; Bishop A. C. Quantitation of Autoinhibitory Defects in Pathogenic SHP2 Mutants by Differential Scanning Fluorimetry. Anal Biochem 2023, 680, 115300. 10.1016/j.ab.2023.115300. 37659706 PMC10530186

[27] Kim S. H.; Bulos M. L.; Adams J. A.; Yun B. K.; Bishop A. C. Single Ion Pair Is Essential for Stabilizing SHP2’s Open Conformation. Biochemistry 2024, 63 (3), 273–281. 10.1021/acs.biochem.3c00609. 38251939 PMC12762099

[28] Cunnick J. M.; Mei L.; Doupnik C. A.; Wu J. Phosphotyrosines 627 and 659 of Gab1 Constitute a Bisphosphoryl Tyrosine-Based Activation Motif (BTAM) Conferring Binding and Activation of SHP2. Journal of Biological Chemistry 2001, 276 (26), 24380–24387. 10.1074/jbc.M010275200. 11323411

[29] Myers M. G.; Mendez R.; Shi P.; Pierce J. H.; Rhoads R.; White M. F. The COOH-Terminal Tyrosine Phosphorylation Sites on IRS-1 Bind SHP-2 and Negatively Regulate Insulin Signaling. Journal of Biological Chemistry 1998, 273 (41), 26908–26914. 10.1074/jbc.273.41.26908. 9756938

[30] Marasco M.; Berteotti A.; Weyershaeuser J.; Thorausch N.; Sikorska J.; Krausze J.; Brandt H. J.; Kirkpatrick J.; Rios P.; Schamel W. W.; Köhn M.; Carlomagno T. Molecular Mechanism of SHP2 Activation by PD-1 Stimulation. Science Advances 2020, 6 (5), eaay4458. 10.1126/sciadv.aay4458. 32064351 PMC6994217

[31] Van Vlimmeren A. E.; Tang L. C.; Jiang Z.; Iyer A.; Voleti R.; Krismer K.; Gaublomme J. T.; Jovanovic M.; Shah N. H. Proximity-Labeling Proteomics Reveals Remodeled Interactomes and Altered Localization of Pathogenic SHP2 Variants. March 1, 2025. 10.1101/2025.02.26.640373. PMC12894930 41429940

[32] Paardekooper Overman J.; Yi J.-S.; Bonetti M.; Soulsby M.; Preisinger C.; Stokes M. P.; Hui L.; Silva J. C.; Overvoorde J.; Giansanti P.; Heck A. J. R.; Kontaridis M. I.; Den Hertog J.; Bennett A. M. PZR Coordinates Shp2 Noonan and LEOPARD Syndrome Signaling in Zebrafish and Mice. Molecular and Cellular Biology 2014, 34 (15), 2874–2889. 10.1128/MCB.00135-14. 24865967 PMC4135572

[33] Müller P. J.; Rigbolt K. T. G.; Paterok D.; Piehler J.; Vanselow J.; Lasonder E.; Andersen J. S.; Schaper F.; Sobota R. M. Protein Tyrosine Phosphatase SHP2/PTPN11 Mistargeting as a Consequence of SH2-Domain Point Mutations Associated with Noonan Syndrome and Leukemia. Journal of Proteomics 2013, 84, 132–147. 10.1016/j.jprot.2013.04.005. 23584145

